# Editorial: One health approach to improve food safety

**DOI:** 10.3389/fmicb.2023.1269425

**Published:** 2023-08-22

**Authors:** Fereidoun Forghani, M. Y. Sreenivasa, Byeonghwa Jeon

**Affiliations:** ^1^IEH Laboratories and Consulting Group, Lake Forest Park, WA, United States; ^2^Department of Studies in Microbiology, University of Mysore, Mysuru, India; ^3^Division of Environmental Health Sciences, School of Public Health, University of Minnesota, Minneapolis, MN, United States

**Keywords:** food safety, one health, foodborne pathogens, *Salmonella*, *Campylobacter*

One Health is a holistic approach that recognizes the association of humans, animals, and the environment to address health challenges (King et al., 2008). Applying One Health principles is becoming increasingly important to address complicated food safety issues that involve various factors from farm to fork (Kniel et al., 2018). The approach usually involves collaboration between public health, veterinary medicine, and environmental health experts to identify and mitigate risks associated with foodborne pathogens. One key aspect of the One Health approach to improving food safety is the understanding that foodborne pathogens can be transmitted through multiple pathways from animals and food production and processing environments. Major foodborne pathogens, such as *Salmonella* and *Campylobacter*, originate from animals, are prevalent in farm environments, and are introduced to the food supply systems. The One Health approach can help understand multiple pathways of foodborne pathogen transmission and identify and address risks at each stage of the food production chain. Our Research Topic published several articles demonstrating the application of the One Health approach to improving food safety.

Whole genome sequencing is frequently used to characterize the virulence factors of foodborne pathogens. *Salmonella enterica* subsp. *enterica* serovar Cerro is rarely found in human cases of salmonellosis but is commonly found in cattle without clinical signs of illness in the United States. Cohn et al. identified genomic features associated with its virulence attenuation in humans. The study found that most *S*. Cerro isolates represent a monophyletic clade within section Typhi and predominantly share a common ancestor with cattle isolates. Specific genomic features associated with *S*. Cerro's infrequent isolation from humans and its adaptation to cattle were identified, which has broader implications for understanding host adaptation in *Salmonella*.

Ramadan et al. studied the *Listeria innocua* strains isolated from milk and dairy products through whole-genome sequencing in search of antibiotic resistance and virulence genes. Gene transfer between microorganisms has been getting increased attention as our knowledge of molecular biology keeps expanding. In this context, some of the microorganisms capable of gene transfer may transfer properties such as virulence between them. This phenomenon can cause major food safety concerns and needs further research. *L. innocua* is generally known as a non-pathogenic *Listeria*. Most importantly, in their work, they found 13 virulence genes involved in the pathogenicity of *L. monocytogenes* in these isolates. Given the possibility of gaining pathogenicity via gene transfer from *L. monocytogenes*, such genetic changes should be under surveillance, and improvements in the development of ultimate preventive control measures are necessary.

The approach should also actively seek to develop interventions to control foodborne pathogens using antibiotic alternatives. Sreepathi et al. isolated a *Levilactobacillus brevis* (RAMULAB52) from fermented *Carica papaya* L. and studied its probiotic properties. Through biochemical assays, autoaggregation testing, gastric juice tolerance assay, adhesion to epithelial cells, etc., it was concluded that this strain possessed the properties necessary to be considered a potential probiotic. Moreover, it showed significant inhibition of α-amylase (86.97%) and α-glucosidase (75.87%) enzymes which can be beneficiary to diabetic patients. Thus, this microorganism is a good candidate to be further studied for application as a probiotic.

Du et al. investigated the effects of a combination of organic acids on acute campylobacteriosis. They showed that the combinatory organic acid treatment improved the clinical outcome of the infection and reduced inflammatory sequelae in the colon. Mice from the combination cohort had lower numbers of immune cells and pro-inflammatory cytokine secretion in their colonic mucosa and lamina propria. The anti-inflammatory effects were not restricted to the intestinal tract but could also be observed systemically. They suggest that oral application of a combination of organic acids could be a promising therapeutic strategy for acute campylobacteriosis without relying on antibiotics.

A study done by Mousavi et al. examined the use of lemon and coriander essential oils as therapeutic agents against *Campylobacter jejuni* infections. The preclinical *in vivo* study found that the essential oils lowered gastrointestinal *C. jejuni* colonization and alleviated clinical signs of acute murine campylobacteriosis. The treatment also reduced pathogen-induced colonic epithelial cell apoptosis and lowered the numbers of macrophages/monocytes and T lymphocytes. The study suggests that the prophylactic application of these essential oils could be a promising measure to prevent severe campylobacteriosis and improve food safety. The use of essential oils as an alternative can achieve the goals of a One Health approach to improving food safety.

Another important aspect of the One Health approach to microbial food safety is the use of surveillance systems to identify and address the source of foodborne illnesses and outbreaks. This allows for early detection of outbreaks and can help identify emerging trends in pathogen prevalence. A study by Kongant et al. demonstrated the usefulness of a new bioinformatics tool called bettercallsal for metagenomic outbreak response workflow. It accurately identifies multiple *Salmonella* serovars from metagenomic or quasi-metagenomic data sets with high accuracy, allowing these isolate-independent methods to be incorporated into surveillance and root cause investigations. The tool was tested against an *in-silico* benchmark data set and on previously well-characterized and sequenced non-selective primary and selective enrichments of papaya and peach samples from separate outbreak investigations. The study suggested that bettercallsal could be a promising tool for *Salmonella* subtyping analysis.

Food safety is a complex issue that requires collaborative efforts from experts in multiple fields. The One Health approach can offer a comprehensive and collaborative approach to improving food safety, helping reduce the incidence of foodborne illnesses, and improving public health outcomes.

## Author contributions

FF: Writing—review and editing. MS: Writing—review and editing. BJ: Writing—review and editing, Writing—original draft.
